# Respiratory Allergies: A General Overview of Remedies, Delivery Systems, and the Need to Progress

**DOI:** 10.1155/2014/326980

**Published:** 2014-03-12

**Authors:** Giuliano Molinari, Giselda Colombo, Cinzia Celenza

**Affiliations:** ^1^Biochemistry Consulting Service, Giuliano Molinari, 20017 Rho, Milan, Italy; ^2^Allergy and Immunology Unit, San Raffaele Hospital, 20132 Milan, Italy; ^3^Quality Assurance Service, Sandoz S.P.A., 21040 Origgio, Varese, Italy

## Abstract

The spread of respiratory allergies is increasing in parallel with the alarm of the scientific community. Evidently, our knowledge of the onset mechanisms of these diseases and, as a consequence, of the available remedies is inadequate. This review provides a brief, general description of current therapeutic resources and the state of research with regard to both drugs and medical devices in order to highlight their limits and the urgent need for progress. Increasing the amount of basic biochemical research will improve our knowledge of such onset mechanisms and the potential efficacy of therapeutic preparations.

## 1. Introduction

It is known that allergic rhinitis (AR) is mainly induced by an IgE-mediated response and shares many features with allergic asthma (AA). AR is often associated with sinusitis or other comorbidities such as conjunctivitis [1–4] and precedes AA. AR and AA not only have a common biochemical onset but, to some extent, also have common remedies. The interdependence between morbidities of the upper and lower airways is now known under the concept of “united airways” and the need for the concomitant treatment of these diseases is recognized.

The IgE-mediated response is not a unique mechanism of allergic reaction onset; other less known mechanisms exist. In fact, five years ago, the ARIA group of experts wrote [5] “*allergen-specific IgE, synthesized in response to allergens in the environment, becomes fixed to Fc*ε*RI on the membranes of mast cells and basophils; this aggregation results in the production of mediators (histamine, leukotrienes and others) that produce the allergic response; however a direct non-IgE-dependent mechanism also exists and the relative importance of non-IgE to IgE-mediated mechanisms is undetermined.*”

At present, we know somewhat more [6–21] and research is proceeding in many directions. Long-term birth cohort studies are underway [22] to assess both the genetic and environmental determinants of allergic responses. Several guidelines are available for the prevention, diagnosis, and therapy of these diseases [23–26], but despite the considerable effort made in studying new remedies, which are proposed in many different pharmaceutical forms as described in the central part of this review, the number of allergic patients is growing, especially with respect to children and young adults.

As a consequence, the need to make progress is increasingly evident. In the last two years, several proposals/requests have been presented with respect to research, the development, regulation, and utilization of therapeutic resources for respiratory allergies [27–35]. Among them, the “call to action” of the European Federation of Allergy (EFA) is probably the most recent and alarming [36]. Since these proposals/requests are considerable in number and very heterogeneous, they are recalled and grouped together at the end of this review. They may be of great interest to allergists, respiratory specialists, pharmaceutical scientists, and manufacturers in the short term as proposals for the improvement of patient management and regulatory modifications or in the medium term for formulation improvements, innovative devices, or diagnostic tests. The authors' opinion on the importance of biochemical research improvement is highlighted in the conclusion. In fact, although the fundamental, biochemical origins of these diseases are known [14–17, 37, 38], better knowledge is required of their basic pathophysiologic pathways and mechanisms [20, 32], which appear to offer the best targets for effective therapy.

## 2. Pharmacological Treatments for Respiratory Allergies

It is known that besides the basic, obvious, but, in some circumstances, difficult-to-follow rule of “avoid contact with allergens,” guidelines suggest many different treatments for adults, while there are tables for disease diagnostic classification and control assessment. However, treatments for children and for women during pregnancy and when breastfeeding are only sometimes described. Remedies are available in both systemic and topical form; they can be preventive as well as curative but are more often symptomatic.

Existing therapeutic preparations can essentially be divided into the following three groups, in which drugs for inflammation reduction belong to the second, while drugs for the recovery of the immune balance belong to the other two:

- preparations for allergen specific immunotherapy,
- traditional symptomatic drugs, and
- anti-IgE biological agents.

Unfortunately, none of these treatments ensure a full recovery from the illness, the causes of which are still partly unknown.

### 2.1. Allergen Specific Immunotherapy

A patient's hypersensitivity can be reduced by a desensitization or hyposensitization treatment known as allergen specific immunotherapy (SIT), which consists of gradual vaccination with progressively larger doses of an allergen. It relies on the progressive skewing of IgG4 antibody production, which is known as a “blocking antibody” due to its ability to compete for the same epitopes as IgE, thus preventing IgE-dependent allergic responses [39, 40].

SIT, in its subcutaneous immunotherapy (SCIT) and sublingual immunotherapy (SLIT) forms, is recognized as an effective treatment for respiratory allergies [41]. It requires the regular administration of allergens over a period of about four years and is thus a consistent expense. If correctly administered, immunotherapy leads to a 20–40% reduction in symptoms that lasts for around eight years after the therapy has ended [42, 43]. Meta-analyses have confirmed the efficacy of the treatment in allergic rhinitis in children [44] and asthma [45]. While treatments with traditional drugs only influence the symptoms, SIT has been shown to have the capability to both cause disease-modifying changes to the underlying atopic condition to prevent new allergic sensitization and arrest the progression of allergic rhinitis to asthma [46]. Nevertheless, SIT must be considered on a case-by-case basis, especially in asthmatic patients, and is only indicated for mild and moderate but not severe asthma because of the risk of anaphylactic reactions. In addition, SIT should not be considered as an alternative, but as complementary to pharmacological therapy, and it is mandatory to start this treatment when asthma is well-controlled by drugs.

SLIT is an orally administered therapy that takes advantage of oral immune tolerance to nonpathogenic antigens such as foods and resident bacteria. While SCIT [47] is used worldwide, SLIT has only been introduced more recently [48]. It is approved in the EC and many other countries [29, 34, 48, 49] but has still not been approved in the USA by the FDA [50]. However, SLIT is gaining support among traditional allergists [13] in the United States, where a solution from allergen extracts can be prepared and administered directly. SLIT drops or tablets have been associated with fewer allergic reactions than SCIT shots. On the other hand, SCIT is thought to improve symptoms more than SLIT. The available comparative studies are still limited in number [51–56], while new cost-effective analyses (CEA) are current at the present time and the debate is ongoing.

### 2.2. Traditional Drugs

Currently available medication options for the treatment of symptoms of respiratory allergies are summarized in Table 1 and then briefly described.

Traditional drug therapy is indispensable in reducing and preventing symptoms and is extremely important in acute, critical cases. Nevertheless, it is rarely useful in the first phase of AR and is unlikely to modify the natural history of the disease, which can become chronic in nature. Medications are classified according to their use, contents, and route of administration.

#### 2.2.1. Drugs for Allergic Rhinitis

It is known that, generally, six classes of drug and nasal saline are used to treat AR [57]: oral and topical H1-antihistamines, intranasal glucocorticosteroids (INCs), mast cell stabilizers (i.e., cromones), decongestants, anticholinergic agents, and leukotriene inhibitors, also called antileukotrienes. Medications used for AR are typically administered orally or intranasally. The intranasal route allows higher concentrations of the drug to be delivered, thus minimizing systemic side effects [58]. The effects of therapies on rhinitis symptoms are summarized in an interesting table in the BSACI guidelines [59].

*Antihistamines*. First-generation antihistamines (brompheniramine, chlorphenamine, diphenhydramine, hydroxyzine, ketotifen, oxatomide, pheniramine, and pyrilamine) are nonselective because they bind all H1 receptors, including those of the central nervous system and can therefore cause sedation. Nonselective antihistamines have been associated with impaired sleep, learning, and work performance and with motor vehicle, boating, and aviation accidents [60].

The second- and third-generation antihistamines (acrivastine, bilastine, cetirizine, desloratadine, ebastine, epinastine, fexofenadine, loratadine, levocetirizine, mizolastine, olopatadine, and rupatadine) are more selective than their first generation counterparts, because they cross with difficulty the blood-brain barrier to bind central H1 receptors. As a result, sedation is reduced [61]. They have a relatively quick onset of action and a relatively long half-life, allowing for once daily dosing [62, 63].

Several antihistaminic preparations are available on the market: oral antihistamines (tablets and drops), nasal sprays that can act more rapidly than oral preparations, and eye drops. They are often produced in combination with other drugs such as mast cell stabilizers and decongestants. 

*Corticosteroids*. Having a potent anti-inflammatory power, corticosteroids can help to treat allergic reactions by blocking inflammation. Intranasal corticosteroids (INCs) are recommended as the first-line treatment for moderate/severe or persistent allergic rhinitis [64–67]. INCs target the inflammatory mechanism of the early and late phases of allergic processes and are therefore effective in treating most symptoms of AR including congestion, sneezing, rhinorrhea, and nasal pruritus. INCs are considered to be more effective than intranasal antihistamines [68, 69]. In the case of prolonged use, the common collateral effects of intranasal steroids are epistaxis and hypotropia of the nasal mucosa.

According to Sastre and Mosges [70], systemic adverse effects are uncommon with older INCs (triamcinolone, flunisolide, beclomethasone, dexamethasone, and budesonide) compared with oral agents (prednisone and methylprednisolone). The second-generation INC agents currently in use (mometasone furoate nasal spray, fluticasone propionate, ciclesonide, and fluticasone furoate) have favorable pharmacokinetic characteristics that further reduce systemic bioavailability (<1%), so lowering the risk of systemic adverse events.

In addition to intranasal preparations, other formulations of corticosteroid are the eye drops used for the treatment of severe ocular, allergic symptoms. 

*Combinations of Nasal Antihistamine with INCs*. While some publications have reported lack of improvement with antihistamine add-on therapy combined with topical nasal steroids in comparison to monotherapy [71–73], recent studies report faster and more complete symptom controls for the combination of azelastine-fluticasone [74–76]. As a consequence, this particular combination therapy may be soon the treatment of choice for moderate-to-severe AR. 

*Intranasal Mast Cell Stabilizers (Cromones)*. The pyranoquinolone derivatives like cromoglicic acid salts (cromolyn) and nedocromil are intranasal mast cell stabilizers. Cromolyn is generally not as effective as antihistamines or INCs but has been shown to be superior to a placebo in reducing the symptoms of the early phase [66]. These drugs mainly have a prophylactic use [77]. They do not diffuse into the blood and the safety profile is good [78]. 

*Decongestants*. Decongestants are *α*-adrenergic agonists that cause vasoconstriction. They can therefore relieve both edema and congestion of the nasal mucosa but do not alleviate nasal itching, sneezing, or rhinorrhea associated with AR [59].

Intranasal decongestants (oxymetazoline, xylometazoline, hydrocodone, and phenylephrine) are often associated with a corticosteroid or an antihistamine to improve the delivery of these drugs [57]. Rebound swelling of the nasal mucosa and “drug-induced rhinitis,” termed rhinitis medicamentosa, may occur with several days (>10) of use [79]. Similarly, oral decongestants (ephedrine, pseudoephedrine, phenylephrine, and phenylpropanolamine) can be found in association with other drugs. They may sometimes produce serious systemic, adverse effects: tachycardia, hypertension, dizziness, insomnia, headaches, sweating, and tremors [78]. 

*Anticholinergic Agents*. Ipratropium is an intranasal anticholinergic agent that blocks muscarinic acetylcholine receptors, thereby inhibiting the mucous secretions within the nasal mucosa. It does not affect sneezing or nasal obstruction [59]. Systemic absorption is minimal, which prevents undesired side effects. Nevertheless, cautious use is advised for patients with narrow-angle glaucoma, prostatic hypertrophy, or bladder neck obstruction, particularly, if another anticholinergic is coadministered by another route [57]. 

*Leukotriene Inhibitors*. Leukotriene inhibitors (montelukast and zafirlukast) are cysteinyl leukotriene 1 (CysL1) receptor antagonists. They are oral agents with a once-a-day recommended intake. Leukotrienes are able to relieve allergy symptoms and inflammation by reducing vasodilatation, mucus secretions, and chemoattraction towards eosinophils [66]. Leukotriene inhibitors appear to be more effective for AA than AR but can also be used to treat the latter [58]. 

*Nasal Saline*. Evidence shows that nasal saline is beneficial in treating nasal AR symptoms [57], particularly, during pregnancy, and in children and in patients who are run down, as it is associated with few adverse effects.

#### 2.2.2. Drugs for Allergic Asthma

Drugs for the treatment of asthma are generally classified as either “controllers,” which are taken daily on a long-term basis to prevent exacerbations by keeping a check on allergic inflammation, or “rapid relievers,” which are taken on demand for rapid relief in cases of abrupt worsening of symptoms [80].

The following description of medications for asthma has been updated in accordance with the GINA guidelines, 2012 ed. [26]. 

*(1) Controller Medications*

*Inhaled Corticosteroids (ICSs)*. Inhaled corticosteroids (first generation: triamcinolone, flunisolide, and beclomethasone; and second generation: budesonide, ciclesonide, fluticasone, and mometasone) are considered to be the most effective anti-inflammatory medications available for the treatment of persistent asthma [81]. Low-dose ICS monotherapy is recommended as first-line maintenance therapy for most asthmatic patients [26, 82].

Since ICSs do not “cure” asthma, most patients will require long-term, if not life-long, ICS treatment. When ICS therapy is unsuccessful in achieving asthma control, add-on therapy with another class of controllers is preferred over increasing the ICS dose. Systemic adverse effects can be associated with higher doses of ICSs, as described below in the reliever medication section. The most common local adverse events associated with ICS therapy are oropharyngeal candidiasis and dysphonia. Mouth washing after each inhalation and/or the use of a spacer device can, however, help to reduce the risk of these side effects [10]. 

*Leukotriene Inhibitors*. The leukotriene inhibitor group includes the 5-lipoxygenase inhibitor (zileuton) and cysteinyl leukotrienes (montelukast, zafirlukast, and pranlukast). They are effective for the treatment of mild or moderate asthma and are generally considered to be safe and well-tolerated. However, patients may be “responders” or “nonresponders” to these agents. Leukotrienes are less effective than ICS treatment when used as a monotherapy; they are prescribed in monotherapy only when the patient is unwilling or unable to use ICSs. Leukotriene inhibitors can also be used as an add-on therapy to reduce the posology of corticosteroids, although they are considered to be less effective than LABAs for this purpose [26]. 

*Inhaled, Long-Acting Bronchodilators (LABAs)*. Inhaled, long-acting *β*_2_-agonists (LABAs) include formoterol, salmeterol, and possible new, once-daily active principles called ultraLABAs [83]. UltraLABAs are indicated for COPD but also have a potential use for asthma. LABAs should not be used as a monotherapy in patients with chronic persistent asthma, because they do not reduce airway inflammation. They also cause *β*_2_ receptor tachyphylaxis, which allows abuse that is associated with an increased risk of morbidity and mortality. LABAs are only recommended when used in combination with ICS therapy, and the possibility of “nonresponder” patients also exists, which is a condition that is genetically determined [84]. 

*Combinations of a LABA and an ICS*. The combination of a LABA and an ICS has been shown to be highly effective in reducing asthma symptoms and exacerbations and is the preferred treatment option in patients whose asthma is inadequately controlled on low-dose ICS therapy. Although there is no apparent difference in efficacy between ICSs and LABAs given in the same or separate inhalers, combinations of ICS/LABA inhalers are recommended because they preclude the use of a LABA without an ICS, are more convenient, and may enhance patient adherence [10]. Combinations of ICS/LABA inhalers, such as salmeterol/fluticasone propionate, budesonide/formoterol, mometasone/formoterol, and beclomethasone/formoterol, are common [26]. Once good control of asthma symptoms is achieved, the guidelines suggest continuing with ICS therapy alone. 

*Long-Acting Muscarinic Antagonists (LAMAs)*. Tiotropium bromide, a muscarinic antagonist, has been proposed as add-on for adults with uncontrolled asthma. It has been shown to improve lung function, but not symptoms [26]. 

*Theophylline*. Theophylline, which is also known in a more soluble form as aminophylline, is an oral traditional bronchodilator with phosphodiesterase 4 (PDE4) inhibitory power and modest anti-inflammatory effects. It is available in sustained release formulations in addition to other active principles. Given its narrow therapeutic window and frequent adverse events (e.g., gastrointestinal symptoms, loose stools, seizures, cardiac arrhythmias, nausea, and vomiting), its use is generally reserved for patients whose asthma is uncontrolled despite an adequate trial of ICSs, LABAs, and/or leukotriene modifiers [26, 82]. 

*Mast Cell Stabilizers (Cromones)*. The efficacy of cromones (sodium cromoglycate and nedocromil sodium) for the long-term treatment of asthma is limited and their anti-inflammatory effect is poor [26]. 

*(2) Reliever Medications*

*Rapid-Acting Inhaled β*_2_ *-Agonists (SABAs)*. Rapid-acting inhaled *β*_2_-agonists (SABAs) are the preferred medications for the relief of bronchoconstriction, its accompanying acute symptoms, and the pretreatment of exercise-induced bronchospasm. SABAs include albuterol, known also as salbutamol, levalbuterol, terbutaline, fenoterol, and pirbuterol. In addition, one LABA, formoterol, which, unlike other LABAs, has a rapid onset of action, is indicated for use in some cases. SABAs should only be taken as needed for symptom relief. Increased use (i.e., three or more times per week) indicates worsening control and signals the need to reassess treatment to achieve the control of symptoms [10]. 

*Combination ICS-Formoterol*. At present, single inhaler maintenance and reliever therapy (SMART) is gaining attention. It is based on administration with the same device as an ICS-formoterol combination and is given for both maintenance and relief as required. The combination budesonide-formoterol can reduce the risk of severe exacerbations and avoid overreliance on SABA medication [85]. In addition, the SMART treatment with budesonide-formoterol has a favorable risk-to-benefit profile [86]. The combination beclomethasone-formoterol has shown favorable results in patients with moderate-to-severe asthma [87]. 

*Anticholinergic Agents*. Short-acting anticholinergic bronchodilators, such as ipratropium bromide and oxitropium bromide, may also be used as a reliever therapy. Their adverse effects have been described earlier in the equivalent paragraph of the AR section. Since these agents appear to be less effective than inhaled rapid-acting *β*_2_-agonists or SMART, they should therefore be reserved as a second-line therapy [26]. 

*Systemic Corticosteroids*. Systemic corticosteroids, such as intravenous methylprednisolone or oral prednisone/methylprednisolone, are indicated for the acute treatment of moderate-to-severe asthma exacerbations. The prolonged use of steroids (more than two weeks) is associated with osteoporosis, arterial hypertension, diabetes, obesity, cataracts, glaucoma, adrenal suppression, and reduced bone growth and height in children. It should therefore be avoided if possible. Inhalation is the preferred route of administration to reduce adverse effects [26].

### 2.3. Anti-IgE Biological Agents

**Figure 1:**
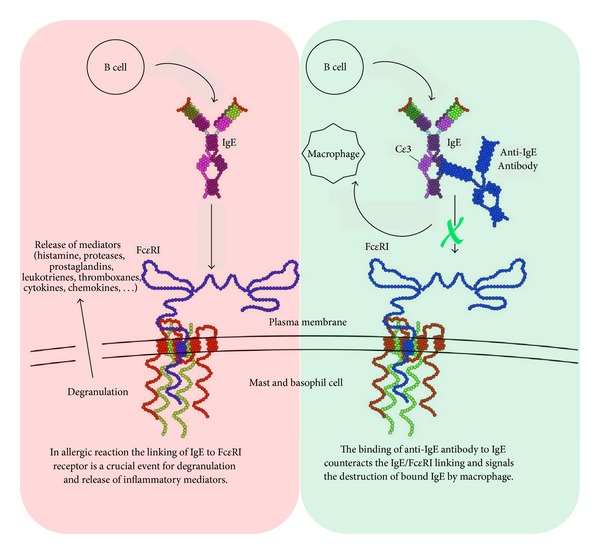
Anti-IgE therapy by monoclonal antibodies (modification of Sari Sabban's image [124]).

Anti-IgE therapy, which is a recent and very promising form of biological therapy, involves the subcutaneous or intravenous injection of monoclonal anti-IgE antibodies. The therapy can be considered a cure in the complete sense of the term, because it counteracts the development of the disease, even before symptoms. Ideally, it should be administered at the first onset of AR to as many patients as possible to reduce AR development and its evolution toward AA. At present, omalizumab is the only approved monoclonal antibody. Omalizumab is a recombinant, humanized, expensive antibody that binds to free and B-cell associated IgE, thus blocking the interaction between IgE and effector cells (Figure 1).

For now, the use of omalizumab is reserved for patients with severe allergic asthma and elevated serum levels of IgE (but not more than 1500 UI/mL, versus normal value <100 UI/mL), whose symptoms remain uncontrolled despite ICS therapy [26]. Omalizumab has a highly selective mechanism of action [88] and a good safety profile, although anaphylaxis has occasionally been reported [89].

Omalizumab reduces symptoms and the frequency of asthma exacerbations by approximately 50%. It has a significantly decreased risk of the hospitalization of patients with uncontrolled severe asthma. The growing interest in anti-IgE therapy in asthma treatment has been highlighted in the PRACTALL guidelines [90].

This very good, but limited, effect of anti-IgE therapy is consistent with the fact that it only prevents IgE mediated stimuli. As the allergic response is triggered by both IgE mediated and non-IgE mediated stimuli, a new therapeutic agent against the latter is required to achieve complete protection. Alternatively, the search for a new therapeutic agent against the totality of stimuli would be an even more ambitious challenge. To achieve such results, pharmaceutical research should identify the possible biochemical steps that are common to the different triggering mechanisms of an allergic response. The existence of such common biochemical steps seems to be highly probable when considering the fact that different stimuli produce, for some aspects, the same final response.

### 2.4. Current Pharmacological Research

In addition to available medications, several new molecular entities are in an advanced phase of clinical study or are in development. Most of them are anti-IL monoclonal antibodies [88, 91] or enzymatic inhibitors, such as phosphodiesterase 4 (PDE4) or phospholipaseA_2_ (PLA2) inhibitors [92, 93], with clearly defined and limited targets. In addition, studies of calcium are continuing, with a particular interest in channel inhibitors [94–97] and FK506 binding proteins [98].

A number of genome-wide association studies (GWAS) have investigated asthma- and allergy-related phenotypes. Results suggest a need to increase pharmacogenetic studies for a better definition of the disease and identification of nonresponder patients [84]. A closer interaction between industry, academia, and health workers is advisable for identifying novel biomarkers linked to well-characterized phenotypes [99].

It is evident that modern molecules are increasingly specific. This narrow approach of pharmacologists presupposes deep and complete knowledge of the complex onset pathway of respiratory allergic diseases in order to achieve the exact identification of the best target for a good pharmacological response. Since this knowledge is unfortunately still incomplete, research is now moving in several different directions in an attempt to identify the best pharmacological target, thus risking a waste of resources.

In order to find the possible biochemical steps common to the different triggering mechanisms of the allergic response, research should take more careful consideration of cytosolic Ca^2+^, as suggested by Ma and Beaven [20]. In fact, in all allergic manifestations, Ca^2+^ mobilization and the subsequent increase in the cytosolic concentration of free calcium [Ca^2+^]_i_ are crucial events [20, 38]. Although findings on the importance of [Ca^2+^]_i_ and its central role in several immunological reactions are not recent discoveries, some of the fundamental biochemical events that may influence its cytosolic concentration remain unclear. For example, it is not precisely known how InsP_3_ produces the Ca^2+^ release from cellular stores and what input originates Ca^2+^ influx and cellular degranulation. Accordingly, a further article will be prepared to provide new hypothetical explanations of some of these unclear events, as well as the possible biochemical steps that are common to the different triggering mechanisms of the allergic response. These unpublished observations will allow pharmacological research to concentrate its efforts in a well-defined, fundamental direction.

## 3. Delivery Systems for Respiratory Antiallergic Drugs

The most common routes of administration of respiratory antiallergic drugs include the preferred oral, transmucosal (nasal, buccal/sublingual, ocular) and inhalation routes, as well as the more invasive injection routes. The drugs come in different pharmaceutical forms such as: tablets, capsules, solutions, suspensions, powders for inhalation and insufflation, solutions for instillation, and injection.

Delivery systems include nebulizers, propellant-based oral and nasal metered-dose inhalers, dry powder inhalers, soft mist inhalers, devices for premetered and device-metered nasal sprays, insufflators, devices for ocular drop instillation, syringes, and accessories such as spacers, facemasks, and needles. This wide variety of dosage forms and devices represents an ambitious challenge for pharmaceutical scientists. Inhalers and sprays in particular have undergone a process of chaotic development in recent years and are continuously evolving. Delivery systems have been reviewed recently [100–103], so only the most commonly used devices will be briefly described here (Table 2). Their major advantages and disadvantages have been clearly tabulated in Lavorini's recent review article [102]. In addition, the article presents recommendations from the Aerosol Drug Management Improvement Team (ADMIT) for inhaler selection, as well as an algorithm for asthma therapy adjustment.

### 3.1. Pulmonary Devices

Pulmonary devices fall into two main categories:

 nebulizers and
 inhalers, which can be divided into three subcategories:
  - propellant-based metered-dose inhalers (pMDIs),
  - dry powder inhalers (DPIs), and
  - soft mist inhalers (SMIs).

#### 3.1.1. Nebulizers (Jet, Ultrasonic, or Mesh Type)

Nebulizers are the oldest inhalation devices. They can be utilized by all patients, including those with weak or slow inhalation capacities or coordination problems like the elderly and children. Nebulizers deliver a cloud of droplets of a drug solution or watery drug suspension. The cloud can be produced in three different ways: air jet, ultrasounds, or, more recently, through a membrane with microholes (mesh). In conventional systems, the cloud is delivered constantly. Nevertheless, the three different principles yield different aerosols with different densities and size distributions at different output rates. Mesh nebulizers have been shown to be more efficient than ultrasound and jet types. In recent years, to reduce environmental aerosol dispersion and increase delivery to patients, jet nebulizers have evolved towards four different subcategories: those with a reservoir tube, those with a collection bag, breath-enhanced jet nebulizers, and breath-actuated jet nebulizers. The breath-enhanced type has two one-way valves to reduce dispersion, while the breath-actuated version only generates aerosol during inspiration. The four subcategories can have consistent differences in delivery, with the reservoir tube type generally being the least efficient and the breath-actuated version the most efficient.

In addition, mesh nebulizers are changing; the last generation models are battery-powered, very light, and silent and give a minimal residual volume. The breath-controlled aerosol delivery (Akita) system, which is an evolution of the adaptive aerosol delivery (AAD) system, is a portable, electronic, vibrating mesh nebulizer that monitors breathing and delivers aerosol only during inhalation. It can be operated in two different breathing modes: normal and slow. A consistent reduction in treatment times is achievable with good deposition by selecting the second operation mode that guides the patient toward deep and slow breathing.

Nebulizers are regulated as medical devices, while the liquid formulations are approved separately and have an “advisable use with” label. For example, suspensions should not be used with ultrasound devices. Nebulizers can be coupled with a facemask or a mouthpiece, with a consequent possible increase in the delivered dose. Mouthpieces are preferable to facemasks because they eliminate losses in the nose and increase deposition in the lungs. Facemasks for nebulizers should have vent holes to reduce deposition on the face and in the eyes.

In conclusion, nebulizers are suitable for all patients, delivering variable doses of a broad range of drugs and not releasing any propellant. Nevertheless, given the high variability in the performance of the different systems, particular attention should be paid to the manufacturer's instructions and the drug label, particularly, when delivering drugs with narrow therapeutic indices. Moreover, nebulizers are often bulky, expensive, require preparation, have a long treatment time (ranging from five to 25 min), and need proper cleaning to avoid contamination.

#### 3.1.2. Inhalers

Inhalers are typically single-patient-use, portable devices that are available in combination with a specific formulation and dose of drug. They have a shelf life of at least 12–24 months and are disposed of when depleted. Unlike nebulizers, inhalers must be developed and approved as drug and device combinations. 

*Propellant-Based Metered-Dose Inhalers (pMDIs)*. PMDIs were the first handheld inhalation devices and were developed in the 1950s. Under the vapor pressure of the propellant contained in the device, they deliver a cloud of droplets of a drug solution or watery drug suspension. Like nebulizers, pMDIs require slow peak inspiratory flow (PIF < 30 L/min) and are therefore suitable for most patients. To ensure reliable dosing, the vapor pressure must be constant all the way through the product's life. With coming into force of the Montreal Protocol, the old chlorofluorocarbon (CFC) propellants have been substituted with hydrofluoroalkane (HFA) propellants, which partly reduce ozone-depleting problems. Usually, pMDIs are triggered by moving a mechanical actuator that opens a metering valve when inspiration begins; another type exists, known as “breath-actuated,” which removes the need to coordinate breathing and drug delivery. The physical characteristics of the sprays delivered by pMDIs favor oropharyngeal deposition and sometimes the consequent development of local irritation or candidiasis. Accordingly, the use of spacers is generally recommended to reduce undesirable oral effects and possibly increase pulmonary deposition. Spacers can be a fixed extension of the device or a separate accessory, and in every case reduce portability, and constitute a problem of adherence for patients. Nonelectrostatic valved holding chambers (VHCs), facemasks, and mouthpieces are other useful accessories, particularly for children [104]. As with nebulizers, it is important to realize that, as well as reducing oropharyngeal loss, all of these accessories and particularly VHCs can increase deposition in the lungs and the delivered dose, with consequent possible therapeutic benefits. On the other hand, there is a risk of delivering the above the upper threshold of the therapeutic window.

In conclusion, pMDIs are portable, robust, cheap, and easy-to-use and are therefore indispensable for disabled patients. However, they are often inefficient and a nonnegligible source of environmental pollution. 

*Dry Powder Inhalers (DPIs)*. DPIs received great consideration after the banning of CFCs and, in a few years, have attained an important position in the market. They do not need propellant, since the drug's release is due to the flow generated within the device by the patient's inspiration effort. Generally, DPIs require only a single inhalation with a medium-high effort and an adequate, ideal flow to produce a 4 kPa pressure drop over the device. Powders are built by aggregating the active principle with a carrier (usually lactose, sometimes mannitol, or others [105]) and are particularly studied in terms of form and cohesion force. Their purpose is to release the active principle under the force of the flow [106]. In addition, deaggregation depends on the internal design of the inhaler, because deaggregation increases when resistance to airflow rises. Considerable differences of resistance are measurable in different inhalers. Like pMDIs, DPIs produce more oropharyngeal depositions than lung depositions, with the exception being high resistance DPIs, which can be used at flow rates <50 L/min. Two categories of DPI exist: premetered and device-metered. In the first of these, the dose is premeasured by the manufacturer as capsules or blisters. In the latter, the device has a reservoir of drug and a control to premeasure the dose. The different preparatory operations of the various devices are a frequent source of patient error.

DPIs do not have propellant and are small, portable, cheap, and breath-actuated. However, the variability of the effective dose with the required medium-high force of inspiration limits their use to patients over five years of age [35]. 

*Mist Inhalers*. Mist inhalers, also known as soft mist inhalers, smart mist inhalers, solution-metering inhalers, or aqueous droplet inhalers, have been introduced in the last ten years. At present, only one model has been approved, although others are being developed. They have different methods of aerosol generation: through nozzles, vibrating mesh, or electrospray. This type of inhaler has two main advantages: the easy-to-use, including patients with a weak or slow inhalation capacity and the absence of propellant, which is replaced by the pushing force of a spring. A mist of droplets of the drug solution is delivered in a slightly longer time than usual, which should help to overcome the problem of synchronization between device actuation and inhalation. Their disadvantages are their size and the fact that they are not inexpensive.

### 3.2. Accessories for Pulmonary Devices

Spacers, valved holding chambers, facemasks, and mouthpieces are common accessories for nebulizers and inhalers. Often, they are dedicated to a particular device. Since spacer devices or facemasks differ in how they deliver a drug, they may not be interchangeable. Moreover, for effective asthma therapy, different age groups require different inhalers [26]. Therefore, the GINA guidelines considered three age groups and suggested six different, alternative devices for children with asthma. Successively, the ICON group has proposed reducing age groups from three to two (<5 years and >5 years) and the number of devices to three [35].

### 3.3. Nasal Devices

Nasal devices fall into three main categories:

 nebulizers,
 sprayers,
  - metered spray pumps and
  - propellant-based nasal sprayers, and
 powder based devices.

#### 3.3.1. Nebulizers

There are relatively few nebulizers specifically designed for intranasal delivery. They work like pulmonary nebulizers but have a nosepiece as an add-on instead of a facemask. A description of them has recently been provided [107].

#### 3.3.2. Sprayers

*Metered Spray Pumps*. Traditional droppers and squeeze bottles are unsuitable for proper dose delivery and are progressively being replaced by multidose metered spray pumps. Typically, these devices atomize and deliver 100 *μ*L of solution or suspension per spray, offering great reproducibility of the emitted dose, spray pattern, and plume geometry. They must contain preservatives to prevent microbial contamination. Nevertheless, the use of preservatives is avoided in more complex systems, which have aseptic air filters or collapsible bag systems included. 

*Propellant-Based Nasal Sprayers*. Propellant-based nasal sprayers are similar to pMDIs.

#### 3.3.3. Powder Based Devices

The powder form is ideal for active principles that are unstable in liquid formulations, do not need preservatives, and can produce longer nasal retention times than liquids. Powder based nasal devices for the treatment of allergies are “snort-actuated” inhalers, similar to DPIs.

Another type of powder device, known as an insufflator, is in development. This device establishes an external, tubular connection between the nostrils according to the principles of Breath-powered Bi-DirectionalTM technology, so that exhalation from one nostril blows the drug into the other and vice versa [107].

### 3.4. Current Research and Development about Delivery Systems

In addition to improving the efficacy of active principles, formulations, and devices, another important objective of pharmaceutical research is improving the relationship between in vitro test data and in vivo behavior. In this direction, the constructive dialogue between industry, regulators, and academic researchers, which started with workshops concerning bioequivalence, is continuing. Some recent, important documents have been published in the last two years. These reflect the official positions of those involved in orally inhaled and nasal drug product (OINDPs) development, with particular attention being paid to their design and analytical control.

With reference to design, physicians complain that, due to the great number of existing devices with different characteristics/instructions, errors are frequent among patients. Clearly, the role of the physician is fundamental in motivating and addressing patients towards the choice of the better device that is compatible with possible individual limitations [108, 109].

The view that, by using the same inhaler, a patient can achieve better control of his/her asthma is emerging [110]. Therefore, the FDA has distributed a couple of draft guidance to device design [111, 112], with the aim of reducing the most common human usage errors. Working documents from the International Medical Devices Regulators Forum (IMDRF) meeting of March 2013 are in the process of being produced.

On July 19, 2012, the association of manufacturers, known as the International Pharmaceutical Aerosol Consortium of Regulation and Sciences (IPAC-RS), presented the IPAC-RS Human Factors webinar [113], with the declared objective being “*to understand and promote best practices for OINDP device design.*”

With reference to analytical controls, three major issues are attracting attention:

- acceptance criteria for materials,
- incorporation of AIM-EDA in the development cycle of orally inhaled products (OIPs), and
- revision of USP.

With reference to the acceptance criteria for materials, in a quality by design approach (QbD), the IPAC-RS last year promoted a series of webinars, the most important [114] of which took place on 11 October, 2012.

With respect to AIM-EDA, there is a proposal by manufacturers to include in pharmacopoeias abbreviated impactor measurement (AIM) and efficient data analysis (EDA) as an alternative approach to the current cascade impaction (CI) for the measurement of aerodynamic particle size distribution (APSD) in product quality assessments [115, 116]. The position of the regulatory authorities appears in Edwin Jao's presentation [117], in which AIM and EDA are cited for the first time.

On the other hand, the same presentation confirmed the authorities' position on delivered dose uniformity (DDU), which is also a matter of debate. Indeed, in June 2011, the Pharmacopeial Forum published an in-process revision of Chapter 〈601〉 and added a new Chapter 〈5〉 by the USP, concerning DDU criteria and particular definitions, which have produced some observations from manufacturers [118, 119]. After that, a revised draft of the US Pharmacopoeia was published by the USP Dosage Forms Expert Committee (EC) on Pharmacopeial Forum [120]. This draft concerns several interesting chapters: 〈5〉 Inhalation and nasal drug products: general information and product quality tests; 〈601〉 Inhalation and nasal drug products: aerosols, sprays, and powder-performance quality tests; 〈602〉 Propellants; 〈603〉 Topical aerosols; and 〈604〉 Leak rate. The deadline for receiving comments was March 31, 2013.

## 4. Need to Progress and Pending Proposals

This general, concise overview of the pharmacotherapy of respiratory allergies has highlighted that, in spite of the availability of new drugs and several specialized devices, in some cases AR, AA, and related comorbidities continue to be uncontrolled diseases that can evolve towards chronicity. Moreover, the levels of these diseases are increasing worldwide.

For this reason, many publications, initiatives, and reports continue to be produced and are a clear expression of the general need to make progress. Some sources containing explicit proposals/requests have been cited above [28, 30, 32, 33, 35, 57, 99, 102, 110–112, 116, 118, 119]. Moreover, the EFA “call to action” presented at the EAACI-WAO World Allergy and Asthma Congress 2013 held in June in Milan emphasizes the role of the pharmacist in identifying allergic patients and calls on policy makers to increase political recognition of respiratory allergies, promote national programmes, and improve access to and reimburse the cost of preventive and/or disease modifying treatments [36].

These explicit proposals/requests are briefly recalled here with regard to the three main directions of outstanding issues: therapy, regulation, and research.

### 4.1. Therapy Improvement

Therapy improvement is invoked through a better use of existing drugs/devices and the better treatment of the comorbidities that negatively influence the control of asthma. A recent review [121] emphasizes the importance of diagnostic tests for the better characterization of patients and the personalization of treatments in childhood AR and AA. The better characterization and identification of responders and nonresponders to targeted asthma treatments have also been suggested for adults, especially with biologics that are costly [84, 99]. Therefore, particular requirements are

- improvement of the treatment strategy with respect to specific patient phenotypes in pediatric [35, 122] and adult asthma [99] in order to identify groups of patients who are susceptible to specific forms of treatment and those at risk of adverse therapy effects [84],
- better patient education and control to achieve optimal adherence to therapy and a health-related quality of life [28, 102],
- better human factor testing and the improved usability of devices to reduce using errors and injuries from medical devices [111],
- prescription of the same device for both ICS and reliever therapy to increase asthma control [85–87, 110], and
- improvement of asthma comorbidity treatment not only for AR or rhinosinusitis but also for other conditions like obesity, heart disease, and COPD (in smokers). For a more complete list of comorbidities, please see Boulet [123].

### 4.2. Regulation Improvement

Regulation improvement by rationalizing and harmonizing regulatory documents and guidelines includes

- revising the USP [118–120] and possibly other pharmacopoeias,
- updating the regulatory aspects of immunotherapy [34], and
- modifying the ARIA two-point classification of AR as “mild” or “moderate/severe” to improve the assessment of AR control [33].

### 4.3. Research Improvement

Research improvement, particularly in diagnostics, device design, analytical control, and basic mechanisms includes

- new biomarkers and new diagnostic tests for the better characterization of patients and the personalization of treatments in both AR and AA [32, 99, 121],
- further studies about the prevalence of local allergic rhinitis (LAR) and improvement of diagnostic methods to better identify patients affected by LAR [30],
- improvement of device design and development [112],
- better analytical controls by incorporation of AIM-EDA in the development cycle of OIPs [116], and
- efforts to unveil the basic mechanisms of allergies [32].

## 5. Conclusion

Several of the proposals/requests referred to the above can find an answer in pharmaceutical research. This is especially true for device design improvements, device analytical controls, new biomarkers, and new diagnostic tests. Physicians and health authorities can provide other fruitful answers. Great, foreseeable benefits could be achieved: the better personalization/efficacy of treatments, improved adherence to treatment, and a better quality of life. Nevertheless, it is unlikely that recovery from illness will only be achieved in these ways.

On the other hand, the current therapeutic treatments, which are preventive, curative, and often only symptomatic, display the previously described evident limits in terms of efficacy and/or adverse effects. Indeed, only two of the available treatments have been shown to have the capability to both prevent new allergic sensitization and arrest the progression of these diseases: SIT and anti-IgE, the best of which seems to be anti-IgE therapy, with an approximately 50% level of improvement. Anti-IgE is, however, also the more expensive treatment, and its prescription is therefore limited.

This therapeutic offer, which is not yet completely adequate, and the increase in the spread of respiratory allergies fully justify the alarm of the scientific community. We should undoubtedly enhance our knowledge of these diseases, and especially their first steps, to counteract their spread and to increase the availability and efficacy of specific therapeutic offers.

For this reason, the EAACI/EFA position paper [32] appears to be extremely appropriate at the present time, particularly in the part that is invoking “*research efforts to unveil the basic pathophysiologic pathways and mechanisms*” of allergies. In fact, although the exceptional progress of molecular biology in the last decade has allowed us to discover many important effectors of the complex pathway of the allergic response, unfortunately, there is lack of information about the biochemical characterization of these effectors, their way of interaction, and particularly their chemical connections and reactions.

Accordingly, an improvement in basic biochemical research should be hoped for regarding the early phase of effector cell activation and allergic signal reception and transduction, with particular reference to intracellular reactions and the cytosolic Ca^2+^ balance. This deeper insight into the molecular mechanisms of respiratory allergy onset would lead to better knowledge and considerable improvement in the classification, diagnosis, and therapy of these diseases and could help to identify new, more effective remedies. In addition, it will lead to greater knowledge of the causes of the widespread increase in these allergies in industrialized areas and will enable consequent social benefits to be realized.

## Figures and Tables

**Table 1:** Treatment of respiratory allergies: drug categories and their targets [26, 59].

Category	Target organ	Target symptom/function	Improvement
Drugs taken daily for the reduction of symptoms and disease control			
Antihistamines	Nose	Sneezing, rhinorrhoea, itching, obstruction	Medium
	Lungs	Coughing, wheezing, shortness of breath	Medium
	Eyes	Itching, watering	High
Corticosteroids	Nose	Sneezing, rhinorrhoea, itching, obstruction	High
	Lungs	Coughing, wheezing, shortness of breath	High
	Eyes	Itching, watering	Medium
Leukotriene inhibitors	Nose	Rhinorrhoea, obstruction	Low
	Lungs	Coughing, wheezing, shortness of breath	Medium
	Eyes	Watering	Low
Anticholinergics	Nose	Rhinorrhoea	Medium
as relievers for asthma (second-line therapy)	Lungs	Coughing, wheezing, shortness of breath	Medium
Cromones	Nose	Sneezing, rhinorrhoea, itching, obstruction	Low
	Lungs	Coughing, wheezing, shortness of breath	Low
	Eyes	Itching, watering	Medium
Decongestants (for not more than 10 days)	Nose	Obstruction	Very high
Inhaled long-acting bronchodilators (LABAs)	Lungs	Coughing, wheezing, shortness of breath	High
Combinations LABA + corticosteroid	Lungs	Coughing, wheezing, shortness of breath	High
Long-acting muscarinic antagonists (LAMAs)	Lungs	Lung function	Medium
Theophylline	Lungs	Coughing, wheezing, shortness of breath	Medium
Anti-IgE monoclonal antibodies	Lungs	Coughing, wheezing, shortness of breath	High

Drugs taken on demand for quick relief of asthma exacerbations			
Rapid-acting inhaled *β*_2_-agonists (SABAs)	Lungs	Bronchoconstriction, coughing, wheezing	High
Combinations corticosteroid + formoterol	Lungs	Bronchoconstriction, coughing, wheezing	High
Systemic corticosteroids	Lungs	Bronchoconstriction, coughing, wheezing	High

**Table 2:** Delivery systems for respiratory antiallergic drugs.

Devices for respiratory antiallergic drugs delivery		
Device use	Category	Type
Pulmonary	Nebulizers	Jet
		Ultrasonic
		Mesh
	Inhalers	Propellant-based metered-dose inhalers (pMDIs)
		Dry powder inhalers (DPIs)
		Soft mist inhalers

Nasal	Nebulizers	Similar to pulmonary nebulizers
	Sprayers	Metered spray pumps
		Propellant-based nasal sprayers
	Powder based devices	Similar to pulmonary DPIs

## Abbreviations

AR: Allergic rhinitis
AA: Allergic asthma
LAR: Local allergic rhinitis
IgE: Immunoglobulin E
IgG: Immunoglobulin G
ARIA: Allergic rhinitis and its impact on asthma
GINA: Global initiative for asthma
InsP_3_: Inositol triphosphate
SIT: Allergen specific immunotherapy
INCs: Intranasal corticosteroids
ICSs: Inhaled corticosteroids
LABAs: Long-acting *β*_2_-agonists
LAMAs: Long-acting muscarinic antagonists
SABAs: Rapid-acting *β*_2_-agonists
pMDIs: Pressurized metered-dose inhalers
DPIs: Dry powder inhalers
OINDPs: Orally inhaled and nasal drug products
AIM: Abbreviated impactor measurement
EDA: Efficient data analysis.
